# P2X7 Receptor Blockade Protects Against Acrolein-Induced Bladder Damage: A Potential New Therapeutic Approach for the Treatment of Bladder Inflammatory Diseases

**DOI:** 10.3389/fphar.2021.682520

**Published:** 2021-08-12

**Authors:** Zhinoos Taidi, Tommy Zhou, Kate H. Moore, Kylie J. Mansfield, Lu Liu

**Affiliations:** ^1^ School of Medical Sciences, UNSW Sydney, Sydney, NSW, Australia; ^2^ St George Hospital, UNSW Sydney, Kogarah, NSW, Australia; ^3^ School of Medicine, University of Wollongong, Wollongong, NSW, Australia

**Keywords:** acrolein, urothelium, bladder inflammation, interstitial cystitis, purinergic P2X7 receptor

## Abstract

Inflammatory conditions of the urinary bladder have been shown to be associated with urothelial damage and loss of function. The purinergic P2X7 receptor has been implicated in several inflammatory conditions. The aim of this study was to investigate the role of the P2X7 receptor in acrolein-induced inflammatory damage using the porcine urinary bladder. For this purpose, an *ex-vivo* model of porcine urothelial damage induced by direct instillation of acrolein into the whole bladder lumen was used. To determine the role of the P2X7 receptor, the bladders were pre-incubated with a selective P2X7 receptor antagonist, A804598 (10 μM), for 1 h. The effects of the acrolein-induced urothelial damage on the bladder’s function were assessed by examining the bladder wall contractile response, structure changes, apoptosis, and oxidative stress in the bladder tissues. The acrolein treatment led to significant damage to the urothelium histology, tight junction expression, and contractile responses. Acrolein also induced apoptosis in the mucosa layer. All these acrolein-induced responses were attenuated by pre-treatment with the P2X7 receptor antagonist A804598. Acrolein also significantly induced DNA oxidation in the submucosal layer; however, the P2X7 receptor antagonism did not show any protective effect towards the acrolein-induced oxidative stress. These findings suggested that the P2X7 receptor is involved in the acrolein-induced damage to the urothelium; therefore, the P2X7 receptor antagonists may be a new therapeutic option for the treatment of bladder inflammation.

## Introduction

Cyclophosphamide is an anti-neoplastic agent, mostly used to treat different types of cancers such as breast cancer, ovarian cancer, and lymphoma (Emadi et al., 2009). Cyclophosphamide is also used as an immunosuppressive drug to treat autoimmune diseases, such as lupus nephritis (Bargman, 2009) and rheumatoid arthritis (Teles et al., 2017). The therapeutic use of cyclophosphamide is limited due to side effects (Stillwell and Benson, 1988), including severe urinary bladder cystitis leading to hemorrhagic cystitis (bleeding and inflammation associated with bladder filling). The patients who receive treatment with the anti-neoplastic agent cyclophosphamide suffer from hemorrhagic cystitis of the bladder in 2–40% of cases, which is characterized by bleeding and inflammation associated with bladder filling (Stillwell and Benson, 1988). Hemorrhagic cystitis is believed to be the direct effect of acrolein (Haldar et al., 2014), a highly toxic urinary metabolite of cyclophosphamide that concentrates in the bladder lumen (Cox, 1979) and damages the bladder lining, the urothelium. Acrolein is an alkylating agent which causes highly toxic effects on the cells by several mechanisms such as protein or DNA adduction as well as induction of oxidative stress, leading to apoptosis and cell death (Moghe et al., 2015).

The patients with hemorrhagic cystitis experience bleeding of the bladder mucosa (frank hematuria) and symptoms of urinary frequency, urgency, and dysuria (painful urination) (Hannick and Koyle, 2019). Similar symptoms are also seen in the patients with interstitial cystitis/bladder pain syndrome (IC/BPS). These conditions are characterized by pain, mainly in bladder filling, but the patients also experience urinary frequency, urgency, nocturia, and microscopic hematuria (Abrams et al., 2002). Therefore, regardless of the etiology, hemorrhagic cystitis and IC/BPS share similar symptoms. The treatment of inflammatory diseases of the urinary bladder is mostly based on the relief of symptoms; however, the current treatments are poorly effective (Sant et al., 2003), and a treatment approach based on the fundamental understanding of the conditions that underly the pathophysiology is highly in demand.

The most common finding on cystoscopy and biopsy of the patients with IC/BPS is mucosal damage and hemorrhages (Johansson and Fall, 1990). Inflammation in the IC/BPS patients is associated with disruption of the urothelial cell layer (Eldrup et al., 1983; Said et al., 1989; Parsons et al., 1991; Liu et al., 2015) and increased density of sub-urothelial afferent nerve fibers (Liu et al., 2014). The urothelial inflammation, which is now part of the diagnostic criteria of IC/BPS (Cox et al., 2016; Birder and Andersson, 2018), includes mucosal damage or complete loss of the urothelial layer, including the protective umbrella cells (Eldrup et al., 1983; Said et al., 1989; Parsons et al., 1991; Liu et al., 2015), as well as increased apoptosis of the urothelial cells (Liu et al., 2015). In addition, the absence of the urothelial cell tight junction protein zona occludens-1 (ZO-1) has been reported (Slobodov et al., 2004), which is suggested to be associated with increased urothelial permeability (Graham and Chai, 2006). Infiltration of inflammatory cells such as mast cells has also been observed (Pang et al., 1995a; Pang et al., 1995b; Yamada et al., 2000). Mast cell activation leads to the release of diverse inflammatory mediators, most importantly histamine (Shan et al., 2019), cytokines (Peeker et al., 2000; Martin Jensen et al., 2018), TNF-α (Furuta et al., 2018), reactive oxygen species (ROS) (Chelombitko et al., 2016), and ATP (Mansfield and Hughes, 2014).

Different animal models have been used to study the urinary tract inflammatory diseases. Among them, cyclophosphamide-induced cystitis is of particular interest (Birder and Andersson, 2018; Augé et al., 2020). These *in-vivo* rodent models mimic the condition of acrolein-induced cystitis by either direct instillation of acrolein into the urinary bladder (Bjorling et al., 2007; Guerios et al., 2008) or a single or chronic intra-abdominal injection of cyclophosphamide (Malley and Vizzard, 2002; Juszczak et al., 2010; Boudes et al., 2011; Martins et al., 2012; Augé et al., 2020). Even a single injection of low doses of cyclophosphamide can result in severe urothelial damage and inflammation in the urinary bladder wall (Malley and Vizzard, 2002; Juszczak et al., 2010). The symptoms observed in the cyclophosphamide-treated animals include increased urinary frequency on cystometry testing (Boudes et al., 2011). The observed tissue damage comprises edema of the lamina propria (Golubeva et al., 2014), partial loss of the urothelium, hemorrhages, and inflammatory cells infiltration (Martins et al., 2012). Similarly, the direct intravesical instillation of acrolein into the lumen of the urinary bladder causes a significant increase in the mucosal edema, increased bladder weight, and increased inflammation score on the microscopic examination (Guerios et al., 2008), together with leukocyte infiltration (Bjorling et al., 2007; Merriam et al., 2011) and loss of the urothelium (Bjorling et al., 2007).

The P2X7 receptors have recently been hypothesized to be involved in bladder cystitis including IC/BPS (Taidi et al., 2019) due to their important role in inflammation (De Marchi et al., 2016). It is suggested that, under pathophysiological conditions, a higher concentration of extracellular ATP (Sun and Chai, 2006) causes prolonged activation of the P2X7 receptor, resulting in extreme permeability of the cell membrane to ions (Hattori and Gouaux, 2012) and formation of a large membrane pore. This will allow larger molecules to pass through the cells, promoting apoptosis and cell death (Karasawa et al., 2017). The activation of the P2X7 receptor has also been shown to increase the expression and secretion of ROS (Wang and Sluyter, 2013; Hiramoto et al., 2020), cytokines (Karmakar et al., 2016), and other inflammatory mediators (Lister et al., 2007; Di Virgilio et al., 2017).

The aim of the present study, therefore, is to investigate whether the P2X7 receptor activation is involved in the urinary bladder inflammatory conditions, using an *ex-vivo* model of porcine urothelial damage induced by the direct instillation of acrolein. For this purpose, we have investigated the effects of acrolein on the structure, apoptosis, and oxidative stress, as well as the expression of tight junction protein ZO-1 and contractile protein, alpha smooth muscle actin (α-SMA) in the mucosa layer of the porcine bladder tissues. We have also assessed the effects of the acrolein-induced urothelial damage on the bladder’s function by examining the bladder wall contractile response in an organ bath preparation. Furthermore, we investigated the role of the P2X7 receptor in the above processes using a selective P2X7 receptor antagonist, A804598.

## Materials and Methods

### Animal Sample and Specimen Preparation

The porcine urinary bladders of females (6–9 months old) were freshly collected from a local abattoir and transported on ice to the laboratory and the average transportation time was approximately 1.5 h. Under these conditions, no changes in the macroscopic and microscopic properties were observed. The choice of gender is relevant to the higher prevalence of urinary bladder inflammatory disorders in females than in males (Hickling et al., 2015). Any undesirable connective and fat tissues on the outer surface of the bladder were removed and the bladders were rinsed with carbogenated Krebs-Henseleit solution (containing in mM: NaCl 118, KCl 4.7, NaHCO_3_ 25, KH_2_PO_4_ 1.2, MgSO_4_ 1.2, CaCl_2_ 2.5, and D-glucose 11.7), pH 7.4, supplemented with 1% of antibiotic-antimycotic solution (10,000 units/ml of penicillin, 10,000 μg/ml of streptomycin, and 25 μg/ml of Amphotericin B, GIBCO 15240062).

### The *Ex-Vivo* Model of Urothelial Damage in Porcine Bladders

The whole urinary bladders were placed in 100-ml organ baths containing carbogenated Krebs-Henseleit solution (as above) maintained at 37°C. The ureters of each bladder were closed, and two fine tubes (PTFE teflon tubing ID 0.3 mm, OD 1.5 mm, wall thickness 0.25 mm, Gecko Optical) were inserted into the bladder lumen through the urethral orifice. To mimic the excretion of acrolein in the urine, the lumen of the bladders was perfused, via the inserted tubes for 4 h, with carbogenated RPMI culture media (1,640 medium, Sigma-Aldrich), in the presence or absence of 0.05% acrolein (110221, Sigma-Aldrich), using a peristaltic pump at a speed of 8 rpm. The concentration of acrolein was determined based on our preliminary experiments for optimizing conditions. Out of the three concentrations (0.01, 0.05, and 1%) being tested, 0.05% acrolein produced moderate and relatively uniform damage to the urothelium of the bladder.

To optimize the concentration of the P2X7 receptor A804598 (01,617, Sigma-Aldrich), different concentrations (1, 10, and 100 μM) were tested in the initial experiments. A804598 at 10 μM was used for the subsequent experiments as this concentration generated a microscopically visible protective effect against the acrolein-induced urothelial damage, and its protection level was similar to that generated by 100 μM of A804598, whereas the result from 1 μM was less obvious. The perfusion of A804598 began 1 h prior to the addition of acrolein and it continued for further 4 h. The bladders without the application of A804598 were perfused with RPMI culture media in parallel.

### Histological Processing and Analysis

Following 4 h of perfusion with and without acrolein, also with and without the P2X7 receptor antagonist, a part of the bladders (closer to the dome) were fixed in Zamboni’s fixative overnight. The tissues then were embedded in paraffin. The sections were cut into 4 µm slices and mounted onto poly-L-lysine coated slides which were dewaxed and rehydrated in xylene and graded ethanol solutions, respectively. These tissue sections were used for both hematoxylin and eosin (H&E) staining and immunohistochemistry.

### Evaluation of Tissue Damage

The fixed and rehydrated sections were stained with H&E. The whole slides were scanned with Aperio’s ScanScope XT digital slide scanner and the images of the representative fields were captured at 20× magnification, which were analyzed with ImageScope software; and the degree of damage was graded based on the criteria shown in Table 1. This method of histological evaluation has been proposed previously in different epithelial tissues (Reali-Forster et al., 1996; Erben et al., 2014) and was chosen based on the damage usually seen in the bladder inflammatory conditions. To assess the urothelial and submucosal injury, one mean score (out of 3) was applied to each field of view. Five slides of each treatment and control samples (*n* = 5 pigs per group) have been evaluated, and, for each slide, approximately 20 fields of view were examined. The scores from each field of view were added to obtain a final mean score for the whole slide.

**TABLE 1:** Microscopic scoring method (adapted from Reali-Forster et al., 1996; Erben et al., 2014).

Region of damage	Severity of damage	Morphology	Score
Epithelial	No damage	Epithelium intact	0
	Mild	2 of 4 layers of cells intact	1
	Moderate	1 of 4 layers of cells intact	2
	Severe	No epithelium left	3
Submucosal	Absent	Normal	0
	Mild	Edema	1
	Moderate	Inflammation	2
	Severe	Hemorrhage	3

### Immunohistochemistry of Caspase-3

In order to detect the immunohistochemical features of apoptosis, 3,3′-diaminobenzidine (DAB) staining was performed to detect caspase-3 (a well-known marker for apoptosis (Luo et al., 2020)) on the porcine urinary bladder tissues. The tissues from the *ex-vivo* model were sectioned and mounted on slides as above. The slides were subjected to antigen retrieval with EnVision FLEX Target Retrieval Solution, Low pH (K8005 Concentrate; Dako) and then pre-incubated in 3% hydrogen peroxide for 5 min to reduce peroxidases. Non-specific staining was blocked by goat serum (1:10) for 30 min. The slides were then incubated with caspase-3 antibody (ab2302 Abcam, 1:200) overnight at room temperature. Following washing in Tris-Buffered Saline-Triton X (TBS-TX) (0.1 M, pH = 7.6) for 3 × 10 min, the slides then were incubated with 1:200 of the anti-goat secondary antibody for 2 h followed by washing in TBS-TX for 3 × 10 min. After 1 h incubation at room temperature with avidin-biotin complex (1:200, Vector Laboratories), the slides were stained in DAB-nickel solution (Sigma) until a brown signal developed (approximately 30 s) and counterstained with hematoxylin (Sigma) for 1 min.

The slides were scanned using Aperio’s ScanScope XT digital scanning system (Aperio’s ImageScope). For the immunoreactive quantification analysis, a percentage area calculation was performed using ImageJ at 20× magnification following the method described previously (Varghese et al., 2014). For this purpose, the blue staining was eliminated following conversion to gray scale, and the threshold was applied to highlight positive cells. The area of interest was then selected, and the measurement was done with the following chosen parameters: area, standard deviation, min and max gray value, area fraction, mean gray value, and limit to threshold. An average of percentage area from different captures of the same tissue (*n* = 6 pigs per treatment group, 10 captures per tissue) has been calculated.

### Immunohistochemistry of 8-Hydroxy-2′-deoxyguanosine

The immunohistochemistry of 8-hydroxy-2′-deoxyguanosine (8-oxo-dG) was also performed on the same bladder tissues as above to investigate the DNA oxidation in the treated bladder tissues. The non-specific binding sites were blocked by hydrogen peroxide (3% dilution). The slides were then incubated with mouse monoclonal 8-oxo-dG antibody (ab62623 Abcam, 1:1,000) overnight at room temperature. The slides were washed 3 × 10 min with TBS-TX. The tissue was then incubated with biotinylated goat anti-mouse IgG secondary antibody (ab6788 Abcam 1:600) at room temperature for 2 h, followed by washing in TBS-TX for 3 × 10 min. The detection was performed using DAB visualization. The signal was amplified using 100 μl of VECTASTAIN ABC Kit (Life Technologies 32,020) and was stained with 100 μl of DAB Enhanced Liquid Substrate system (Sigma D3939) until a brown signal developed (approximately 30 s). The samples were then counterstained with hematoxylin (30 s), washed with water (30 s), and then stained with Scott’s blue (30 s).

The slides were then scanned with Aperio’s ScanScope XT digital scanning system and viewed with Aperio’s ImageScope. Randomized 20× magnified images of the urothelium and suburothelium were taken from the scanned slides and analyzed on ImageJ (*n* = 5 per treatment group, 10 captures per slide). The urothelium and the white spaces were measured and cut out to establish the area of interest. The Weka segmentation on ImageJ was used to identify and count the positively stained cells for oxidative damage. The size and circularity parameters were established as 50-infinity and 0.02–1, respectively. The cell count was defined as the number of positively stained cells/area of interest (cells/mm^2^).

### ZO-1 Immunofluorescence Staining

The immunohistochemistry of ZO-1 tight junction protein double labelled with contractile protein α-SMA was performed on the same bladder tissues. The slides were subjected to antigen retrieval, as described above. Then they were then incubated with 10% goat serum for 30 min to block unspecific binding sites of secondary antibody followed by incubation with anti-ZO-1 antibody (Invitrogen 61–7,300, 1:100) and anti-α-SMA antibody (Dako M085129-2, 1:200) overnight at room temperature. Following the incubation with the primary antibody, the slides were washed (3 × 10 min) in TBS and tagged with a secondary fluorescent antibody for 2 h at room temperature (ZO-1; Alexa Fluor 594, 1:200, ab150080, α-SMA; Alexa Fluor 488, 1:200, ab150117). After the secondary antibody, the slides were washed again with TBS (3 × 10 min) and then mounted with DAPI.

The immunoreactive images were captured using the Neurolucida microscope, 40× objectives, and analyzed using ImageJ. ZO-1 scoring was conducted using the criteria in Table 2 at 40× magnification (*n* = 5 for each group). The mean score was determined for each treatment group and the results were expressed as mean ± SEM. The level of α-SMA immunoreactivity in the suburothelium was estimated as strong (+++), moderate (++), and weak (+).

**TABLE 2:** Microscopic scoring method for semi-quantification of ZO-1-IR (adapted from Diezmos et al., 2018).

Criteria	Score
ZO-1-IR widely present on the urothelium	5
Majority of urothelial ZO-1-IR present, but some ZO-1-IR absent	4
ZO-1-IR present on approximately 50% of the urothelium	3
ZO-1-IR on the urothelium limited to less than 25%	2
Majority of urothelial ZO-1-IR absent, but some ZO-1-IR present	1
ZO-1-IR is fully absent from the section	0

### Contractile Responses

Following 4 h of perfusion, each bladder was dissected into 5 × 10 mm strips: intact strips (containing the entire layer of the bladder wall), detrusor strips, and mucosal strips (containing the urothelium and lamina propria). Each strip was suspended with 1 g initial tension in a 3 ml organ bath containing carbogenated Krebs-Henseleit solution at 37°C and was equilibrated for 60 min before the addition of acetylcholine (ACh). There were five different treatment groups: non-treated fresh control; perfusion control; perfused with acrolein (0.05%); perfused with A804598 (10 µM); and perfused with acrolein + A804598 (*n* = 6–9 porcine per treatment group). Discrete concentration-response curves to ACh were constructed using increasing concentrations (from 10^–7^ to 10^–2^ M). Each concentration was left in contact with the tissue until it reached the maximal contractile response before washing. There were 15 min (lower than 10^–4^ M) to 30 min intervals between each concentration to avoid desensitization. The contractility of each strip was registered using a Grass FTO3C force transducer and recorded by Polygraph 3.0 computer program (Mr E. Crawford, UNSW Sydney, NSW, Australia).

### Statistical Analysis

For tissue damage evaluation, the quantitative immunohistochemistry of caspase-3 and 8-oxo-dG and ZO-1 scoring were assessed blindly. The results were statistically analyzed with GraphPad Prism 8. One-way ANOVA was used for multiple group comparisons, and the statistical significance was determined using the Bonferroni post-test where *p* < 0.05 was considered significant. For contractile responses of isolated tissue strips, the results were analyzed by two-way ANOVA, followed by Bonferroni’s multiple comparisons. For all data, the *n* value refers to the number of pigs.

## Results

### Effects of the P2X7 Receptor Blockade on the Acrolein-Induced Morphological Damage of the Bladder Mucosa

**FIGURE 1:**
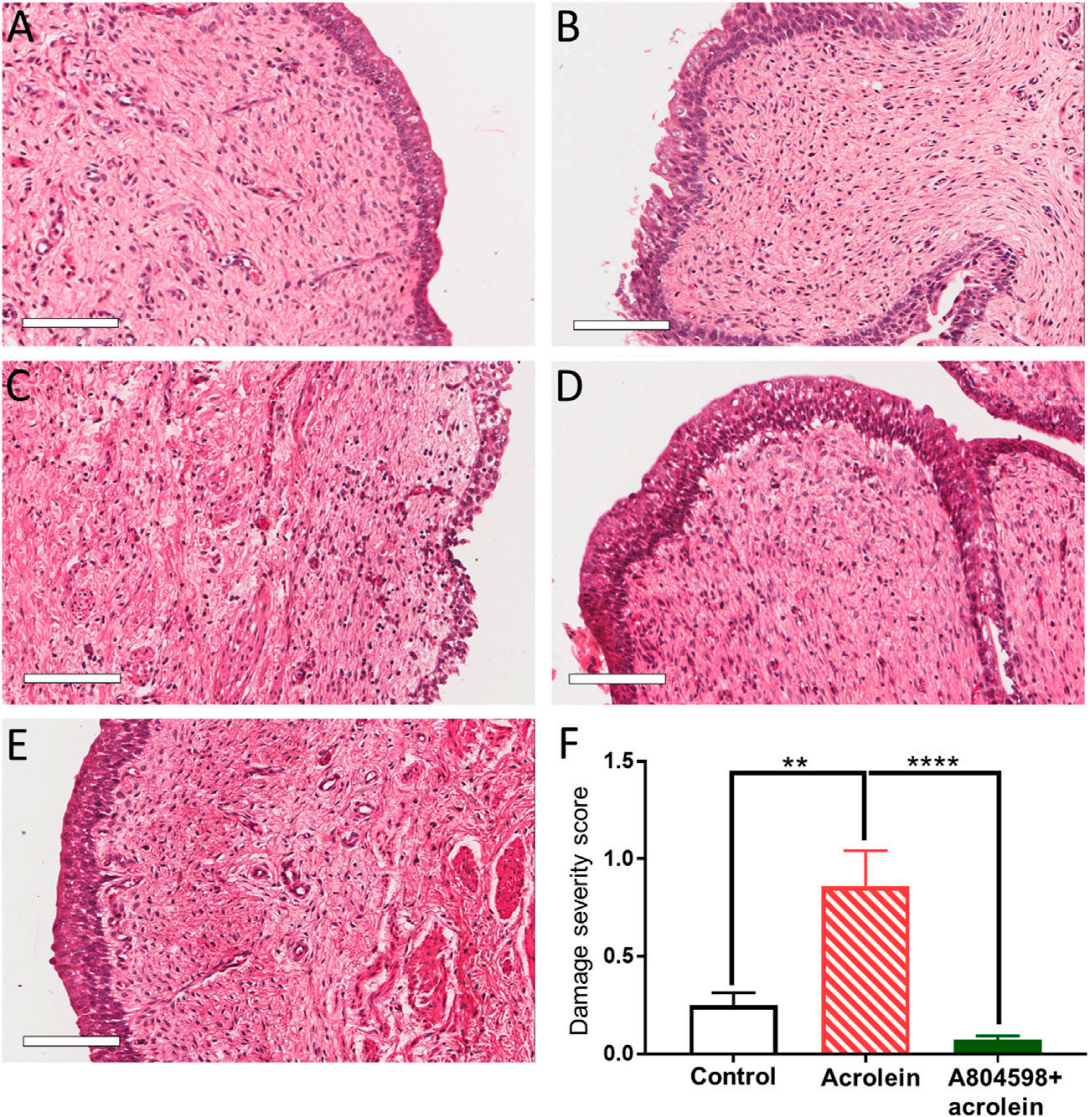
Histological comparison of the porcine bladder mucosal sections of acrolein-induced *ex-vivo* inflammatory model. **(A)** Fresh control: hematoxylin and eosin (H&E) staining of the porcine bladder fixed with Zamboni’s solution immediately following dissection. **(B)** Perfusion control: H&E staining of the porcine bladder which has undergone 4 h perfusion with the RPMI 1640 media before fixation in Zamboni’s solution. **(C)** Acrolein treated: H&E staining of the porcine bladder perfused with acrolein (0.05%) for 4 h. **(D)** A804598 only: H&E staining of the porcine bladder pre-treated with A804598 (10 μM) for 1 h prior to 4 h perfusion with RPMI media. **(E)** A804598 + acrolein: H&E staining of the porcine bladder pre-treated with the P2X7 receptor antagonist A804598 (10 μM) for 1 h prior to 4 h perfusion with acrolein (0.05%). **(F)** Quantitative measurement of the severity of bladder mucosal damage based on the criteria described in Table 1. The data are shown as mean ± SEM from *n* = 5 bladders for each group (**p < 0.01; ****p < 0.0001 compared to the acrolein treated group, one-way ANOVA followed by Bonferroni test). Scale bars = 100 μm.

The H&E staining showed that the perfusion control group maintained the structure of the bladder wall compared to the fresh (non-perfused) control, with only minor damage to the urothelium in some parts (Figures 1A,B). However, following 4 h of acrolein treatment, there was a remarkable level of damage to both the urothelium and the suburothelium (Figure 1C). In the bladders pre-treated with the P2X7 receptor antagonist A804598 (10 µM), the whole mucosa layer (urothelium and suburothelium) was clearly protected from the acrolein-induced damage (Figure 1D). The bladders that were perfused with A804598 alone also maintained their structure (Figure 1E).

To perform the quantitative analysis of the histological evaluation, 20 sections were examined from five bladders of each group (2 different tissue sections from each bladder). Acrolein significantly increased the mucosal damage compared to the perfusion control (Figure 1F, *p* < 0.01, *n* = 5, one-way ANOVA, followed by Bonferroni’s multiple comparisons test). However, the A804598 pre-treatment significantly protected the mucosal layer from the acrolein-induced damage (Figure 1F, *p* < 0.0001, *n* = 5). Although A804598 alone showed some protection against the perfusion-induced damage to the urothelium (Figures 1B vs 1E), the statistical analysis did not show a significant difference between the two groups (data not shown).

### Effects of the P2X7 Receptor Blockade on the Acrolein-Induced Bladder Mucosal Apoptosis

**FIGURE 2:**
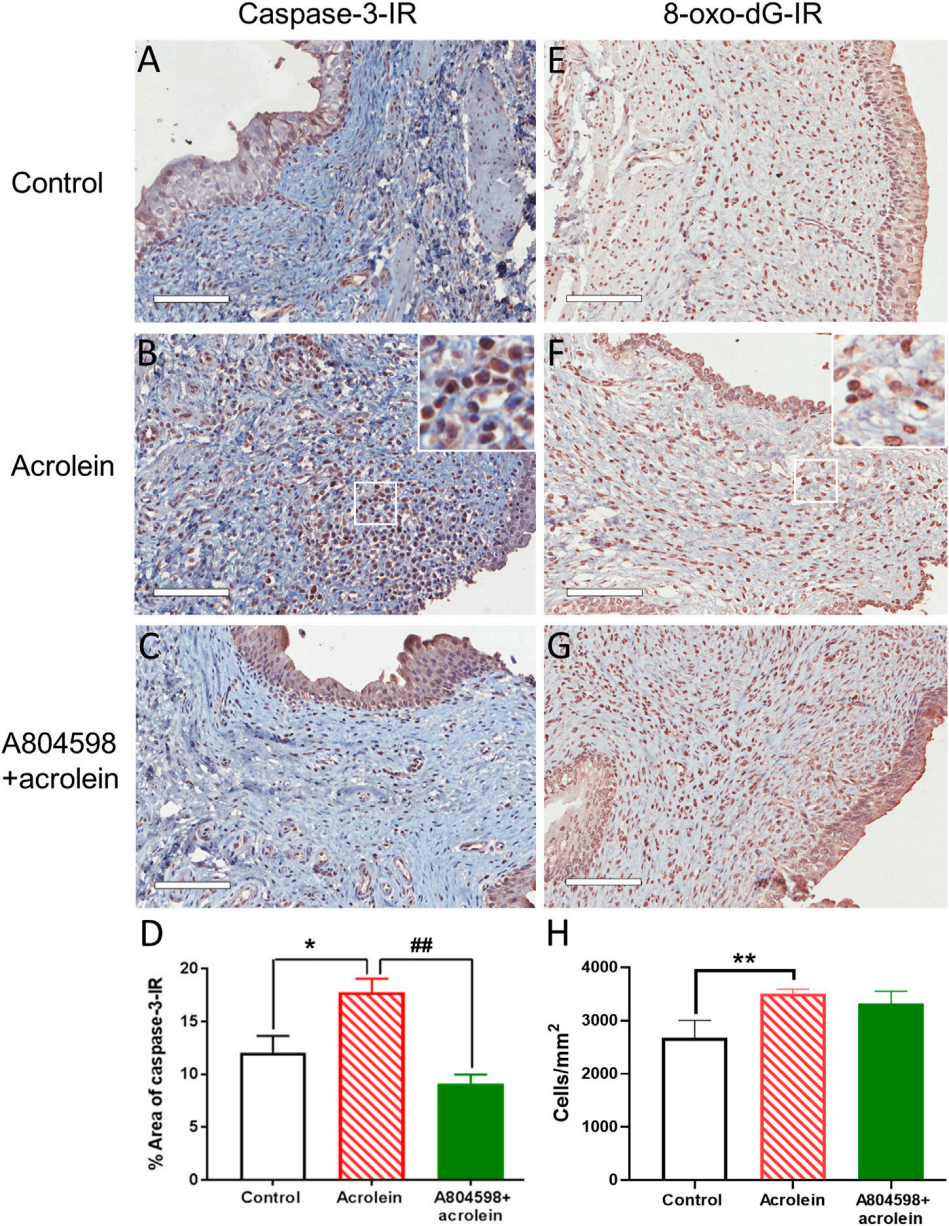
Representative images and quantitative analysis of caspase-3 immunoreactivity and 8-oxo-dG immunoreactivity in the mucosal layer of the porcine bladders. **(A,E)** Perfusion control. **(B,F)** Treated with acrolein (0.05%). **(C,G)** Treated with A804598 (10 μM) and acrolein (0.05%). **(D,H)** Quantitative analysis of caspase-3 immunoreactivity (IR) and 8-oxo-dG-IR, respectively, in the mucosa of the porcine bladders with different treatments using ImageJ. The data are shown as mean ± SEM from *n* = 6 bladders in caspase-3-IR and *n* = 5 bladders in 8-oxo-dG-IR, for each group. Significance was determined by one-way ANOVA with Bonferroni test. *p < 0.05 and **p < 0.01 compared to the acrolein-treated group. Scale bars = 100 μm. High power image inserts are enlargements of regions outlined in **(B,F)**, and many caspase-3-IR and 8-oxo-dG-IR positive cells with brown-stained nuclei are present.

In the perfusion control tissues, weak caspase-3-IR was mainly localized on the urothelium (Figure 2A). In the acrolein-treated bladders, there was a significant increase in the caspase-3 positive cells seen in the urothelial and sub-urothelial regions (Figure 2B). In the bladders co-treated with A804598 (10 µM) and acrolein, caspase-3-IR was greatly reduced, especially in the submucosal region (Figure 2C). The quantitative analysis of caspase-3-IR showed that acrolein-enhanced caspase-3-IR compared to control (*p* < 0.05, *n* = 6, one-way ANOVA followed by Bonferroni test) was significantly blocked by the presence of A804598 (*p* < 0.01, *n* = 6), suggesting that the P2X7 receptor blockade inhibited acrolein-induced apoptosis (Figure 2D).

### Effects of the P2X7 Receptor Blockade on the Acrolein-Induced Mucosal Oxidative Damage

Acrolein treatment visibly increased the density of the cells with positive staining for 8-oxo-dG, a marker of oxidative damage, compared to control (Figures 2E,F). However, the P2X7 receptor antagonist A804598 did not appear to inhibit the action of acrolein (Figure 2G). These observations were confirmed by the quantitative analysis, showing that acrolein (3,507 ± 85 cells/mm^2^) significantly increased the cell count of positively stained cells compared to control (2,485 ± 195 cells/mm^2^, *p* < 0.01, *n* = 5, Figure 2H). However, the combination of acrolein and A804598 did not change the number of oxidative damaged cells (3,322 ± 230 cells/mm^2^) compared to that of those treated by acrolein only.

### Effects of the P2X7 Receptor Blockade on the Acrolein-Induced Reduction of the Expression of ZO-1 and α-SMA

**FIGURE 3:**
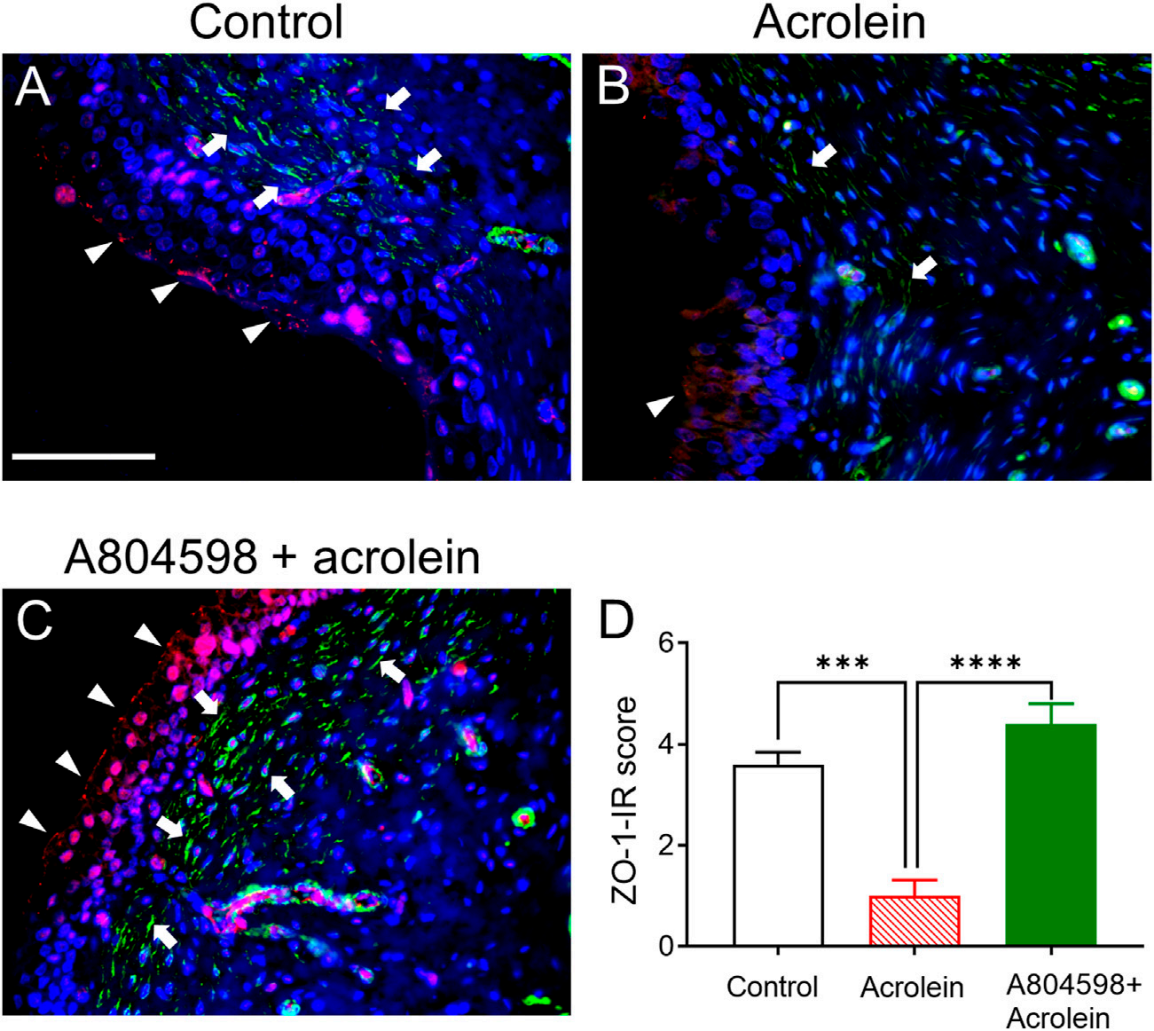
The immunoreactivity (IR) of tight junction protein ZO-1 and α-SMA in the mucosal layer of the porcine bladders. **(A)** Perfusion control: ZO-1-IR (red color) was primarily observed on the luminal side of the bladder urothelium and α-SMA-IR (green color) was localized to the spindle-shaped myofibroblasts in the sub-urothelial region. **(B)** Treated with acrolein (0.05%): the distribution of ZO-1 was disrupted, and it was even totally missing at many parts of the urothelium, and the α-SMA-stained myofibroblasts were greatly reduced. **(C)** Treated with A804598 (10 μM) and acrolein (0.05%). **(D)** Quantitative measurement of ZO-1-IR on the bladder mucosa based on the criteria described in Table 2. The data are shown as mean ± SEM from *n* = 5 bladders for each group (***p < 0.001; ****p < 0.0001 compared to the acrolein treated group, one-way ANOVA followed by Bonferroni test). Scale bars = 100 μm. The arrowheads and arrows designate ZO-1-IR and α-SMA-IR, respectively. DAPI staining (blue color) shows cell nuclei.

The double labelling of ZO-1 and α-SMA on bladder tissues from the *ex-vivo* pig bladders showed that the expression of the tight junction protein ZO-1 on the control bladder urothelium was detectable in the urothelial cell layer (Figure 3A). In the sub-urothelial space, spindle-shaped myofibroblasts were detected by α-SMA staining (Figure 3A). However, the distribution of ZO-1 was disrupted and it was even totally missing at many parts of the urothelium in the acrolein-treated bladders (Figure 3B). Similarly, acrolein treatment has greatly reduced the number of α-SMA-stained myofibroblasts in the sub-urothelial layer (Figure 3B). Pre-treatment with A804598 inhibited the acrolein-induced damage on both ZO-1 in the urothelium and α-SMA expression in the sub-urothelial space (Figure 3C). The ZO-1 immunoreactivity was significantly reduced by acrolein treatment, but the action of acrolein was inhibited by the presence of A804598 (Figure 3D). The ZO-1 expression level in the acrolein plus A804598 group was similar to that in the control (Figure 3D). In the suburothelial layer, altered α-SMA immunoreactivity was observed with weak signals (+) in acrolein-treated tissues and strong signals (+++) in the acrolein plus A804598 group compared to the moderate level (++) in the control.

### Effects of the P2X7 Receptor Blockade on the Acrolein-Induced Reduction of the Contractile Responses of the Bladder Strips to ACh

**FIGURE 4:**
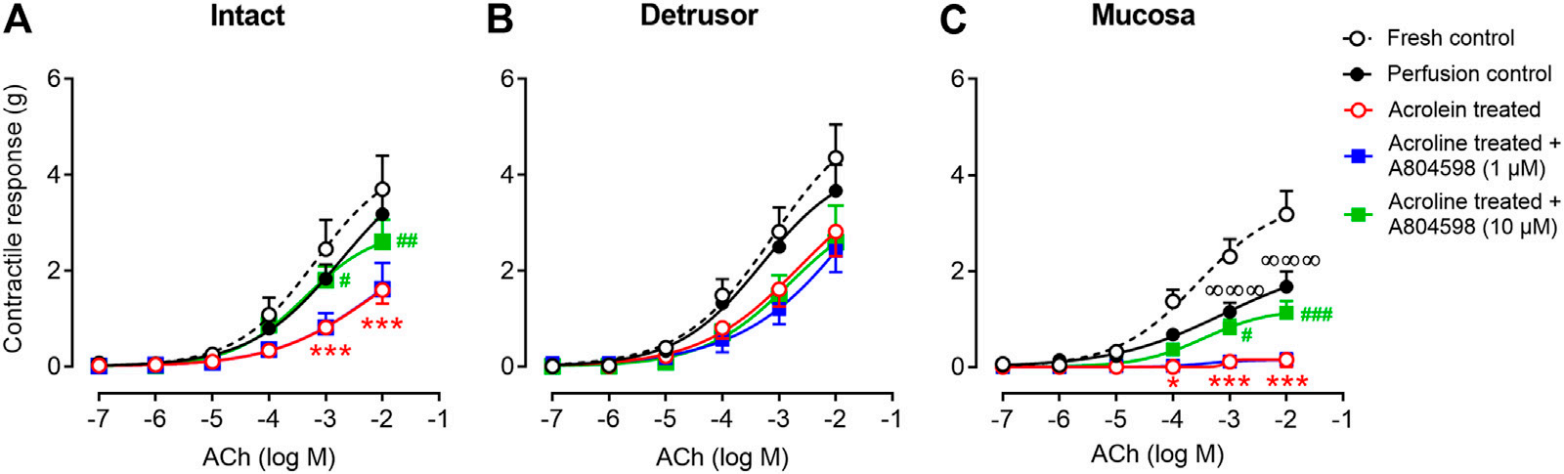
The contractile responses of the porcine bladder strips to ACh. **(A)** Intact strips (containing the entire layer of the bladder wall). **(B)** Detrusor muscle strips. **(C)** Mucosal strips (containing the urothelium and the lamina propria). The contractile responses to ACh were similar in the fresh control and the perfusion control in both the intact **(A)** and detrusor **(B)** strips, but the ACh-induced contraction was reduced in the mucosal strips of the perfusion control **(C)**. In the acrolein-treated bladders, the contractile responses to ACh were significantly diminished in the intact strips and completely abolished in the mucosal strips but were slightly reduced in the detrusor strips. Pre-treating bladders with the P2X7 receptor antagonist A804598 at 1 µM showed no effect but at 10 µM significantly reversed acrolein-induced reduction in response to ACh in the intact and mucosal strips. The data are shown as mean ± SEM from *n* = 6–11 bladders for each group and analyzed by two-way ANOVA, followed by Bonferroni test (^∞∞∞^ *p* < 0.001, compared to the fresh control group; *p < 0.05 and ***p < 0.001, compared to perfusion control; #*p < 0.05,* ^##^ *p* < 0.01, and ^###^ *p* < 0.001, compared to the acrolein-perfused group).

ACh (10^–7^–10^–2^ M) produced contractions of intact, detrusor, and mucosal strips of fresh and perfusion control bladders in a concentration-dependent manner (Figures 4A–C). Following acrolein treatment, the contractile responses to ACh were significantly diminished in the intact strips (Figure 4A) and completely abolished in the mucosal strips (Figure 4C). Acrolein showed a negligible impact on the contractility of the detrusor strips to ACh (Figure 4B). Pre-treatment of the bladders with the P2X7 receptor antagonist, A804598 (10 μM), significantly inhibited the acrolein-induced reduction in the response to ACh in the intact (Figure 4A) and mucosal strips (Figure 4C), but a lower concentration of A804598 (1 µM) did not show effects. The pEC_50_ values were similar among different groups (Table 3).

**TABLE 3:** potency and efficacy of ACh in contracting the porcine bladder strips.

Treatment	Intact		Detrusor		Mucosal	
	pEC_50_	E_max_ (g)^a^	pEC_50_	E_max_ (g)^a^	pEC_50_	E_max_ (g)^a^
Perfusion control	3.30 ± 0.13 (*n* = 9)	3.18 ± 0.47	3.60 ± 0.14 (*n* = 9)	3.66 ± 0.54	3.71 ± 0.21 (*n* = 9)	1.67 ± 0.32
Acrolein treated	3.19 ± 0.15 (*n* = 11)	1.55 ± 0.18**	3.34 ± 0.16 (*n* = 11)	2.82 ± 0.51	ND (*n* = 11)	0.15 ± 0.15***
Acrolein + A804598 (10 μM)	3.45 ± 0.15 (*n* = 6)	2.75 ± 0.30^#^	3.31 ± 0.24 (*n* = 6)	2.59 ± 0.76	3.55 ± 0.19 (*n* = 6)	1.13 ± 0.25^#^

Data represent the mean ± SEM.

a E_max_ values are the contractile responses produced by ACh at the highest concentration used (10^–2^ M).

**p < 0.01 and ***p < 0.001 compared to the perfusion control group.

^#^*p* < 0.05 compared to the acrolein treated group.

ND: could not be determined.

## Discussion

In this study, we have established an *ex-vivo* model of urothelial damage by perfusion of acrolein directly into the porcine bladder lumen. In this model, acrolein induced significant damage to the urothelial structure (urothelial cell loss) and function (contractile response of the bladder mucosa to ACh). This damage mimics that described in the patients with inflammatory bladder conditions (e.g., IC/BPS and hemorrhagic cystitis) (Liu et al., 2015). Acrolein has also induced increased cell apoptosis, decreased urothelial ZO-1 expression, and decreased sub-urothelial myofibroblasts. However, the most important finding of this study was that pre-treatment with a selective P2X7 receptor antagonist, A804598, significantly attenuated the effects of acrolein. Acrolein treatment also triggered a significant increase of oxidative damage in the sub-urothelial cells; however, this was independent of the P2X7 receptor as the P2X7 receptor blockade did not exert any protective effect on the acrolein-induced mucosal oxidative damage.

In this study, we have observed acrolein-induced urothelial and sub-urothelial damage. The urothelium plays an active role in the physiology and pathophysiology of the urinary bladder (Apodaca, 2004). In the IC/BPS patients, the urothelium layer is normally thinner (1–2 cells thick), compared to the urothelium from normal bladders (approximately five cells thick), and, in the IC/BPS patients, there is usually no distinct layer of umbrella cells (Slobodov et al., 2004). Similar disruption and severe damage to the urothelium have also been shown previously in different animal models of interstitial cystitis induced by cyclophosphamide or acrolein (Eser et al., 2012; Martins et al., 2012). An important finding of this study was that the blockade of the P2X7 receptor by its selective antagonist, A804598, protected the urothelium from this structural damage as seen by the immunohistochemistry. This is supported by the finding of the upregulation of the P2X7 receptor in the submucosal layer in a mouse model of hemorrhagic cystitis induced by cyclophosphamide (Martins et al., 2012). Taken together, these findings may suggest that the P2X7 receptor is involved in the process of cyclophosphamide-induced hemorrhagic cystitis seen in the chemotherapy patients (Stillwell and Benson, 1988; Haldar et al., 2014).

Acrolein treatment has also increased apoptosis in the urothelium and the sub-urothelial layer compared to the control group, as shown by the significant elevation in the caspase-3 positive cells. Apoptosis, programmed cell death, can be activated by a diverse range of pathological stimuli (Elmore, 2007). Acrolein is well known to cause cell death by either apoptosis or necrosis via the activation of the caspase cascade (Kern and Kehrer, 2002). Irregular and excessive apoptosis can lead to many pathological conditions (Orrenius, 1995). Increased apoptosis has been detected in the bladder tissues obtained from the patients with different types of urinary tract dysfunction, including IC/BPS and ketamine-induced cystitis (Liu et al., 2015), and it also has been reported previously in a cyclophosphamide-treated rat model of bladder cystitis (Tripathi and Jena, 2010). Therefore, urothelial cell apoptosis and death can be common pathophysiology of the inflammatory bladder conditions, and they all share similar symptoms. This blockade of apoptosis might be an efficient way to interrupt further damage to the urothelium and, therefore, to prevent the development of the symptoms.

Interestingly, our study shows that pre-treatment of the bladder with the selective P2X7 receptor antagonist, A804598, significantly decreased the amount of acrolein-induced caspase-3 positive cells. The P2X7 receptor activation is known to induce apoptosis. In the presence of a high extracellular concentration of ATP, which is highly related to the pathophysiological condition of interstitial cystitis (Sun et al., 2001), the P2X7 receptor can stay activated for a longer time leading to increased intracellular calcium levels (Suadicani et al., 2006) as well as increased cell permeability to larger molecules (Garcia-Marcos et al., 2006) leading to downstream signaling resulting in apoptosis and cell death (Li et al., 2014). The P2X7 receptor expression in the porcine bladder is abundant, with a dense immunoreactivity on the urothelial cells and moderate level on the suburothelial spindle-like cells (likely to be myofibroblasts) and some immune cells (Supplementary Figure 1). Since the P2X7 receptor expression level was not affected by acrolein treatment (Supplementary Figure 2), the acrolein-induced apoptosis did not appear to be associated with P2X7 receptor upregulation. Since severe bladder mucosa damage was seen in the acrolein-treated group, we expected to see a substantial amount of ATP released into the bladder lumen. Surprisingly, the amounts of ATP in the perfusion media were not significantly different between groups (Supplementary Figure 3). This is due to the rapid degradation of ATP by ecto-ATPases which are largely expressed and secreted by epithelial cells. Since we measured the amount of ATP after 4 h perfusion, it cannot be ruled out that the enhanced extracellular ATP due to cell damage had occurred initially, but its action was masked by the ATP degradation. Although our study did not give a clear-cut answer on how the P2X7 receptor is involved in the acrolein-mediated apoptosis, the protective effect by antagonizing the P2X7 receptor demonstrated in the current study was consistent with a study on the liver of septic mice, showing that a selective non-competitive P2X7 receptor antagonist, Brilliant Blue G, decreased apoptosis (Savio et al., 2017; Geraghty et al., 2019). To our knowledge, our study is the first to report a reduction in the acrolein-induced apoptosis by a P2X7 receptor antagonist in the bladder.

8-oxo-dG is a biomarker of cellular oxidative DNA damage (Delaney et al., 2012), and it has been used regularly as an indirect measurement for ROS (Mangal et al., 2009). To investigate the role of oxidative stress in acrolein-induced damage to the urothelial layer, the immunohistochemical quantification of the positively stained cells for 8-oxo-dG was performed. It was found that acrolein treatment significantly increased the cells with oxidative damage compared to control. These results fall in line with other studies demonstrating acrolein-induced oxidative stress and ROS production using different cell types such as PC-12 neuron-like cells (Tian and Shi, 2017) and airway epithelial cells (Yadav et al., 2013). However, our results show no significant reduction in ROS in the cells treated with the P2X7 receptor antagonist. This suggests that the ROS-generating component of acrolein is not mediated through the activation of the P2X7 receptor.

In the present study, we have detected the expression of tight junction protein, ZO-1, on the porcine urothelial cells. Acrolein was clearly associated with the degradation of tight junction protein ZO-1 in the bladder urothelium. The integrity of the tight junction proteins is essential to maintain the urothelial barrier function. Any disruption or decrease in the expression of the tight junction proteins can cause an increase in the urothelial permeability followed by further damage to the underlying layers of the bladder wall by stimuli such as small ions, bacteria, or chemical irritants. Defective expression and function of tight junction proteins, including ZO-1, have been shown previously in the patients with various bladder conditions, for example, IC/BPS and hemorrhagic cystitis (Lee and Lee, 2014). IC/BPS has been termed “leaky” urothelium, which refers to an increased passage of small ions across the urothelial barrier (Eldrup et al., 1983). Aberrant expression of tight junction proteins and increased urothelial barrier permeability may also influence the sensory function of the bladder (Carattino et al., 2013), as high ion contents of urine such as K^+^ can alter neuronal excitability (Medford-Davis and Rafique, 2014), as well as muscle function (Allen et al., 2008).

In the current study, pre-incubation of the bladders with A804598 (10 µM) has shown protection of ZO-1 tight junction protein expression from acrolein-induced damage. Previous studies have supported this notion. Brilliant Blue G maintained the expressions of the tight junction proteins in the lung tissue, in a model of neurogenic pulmonary edema in rats (Chen et al., 2014). Also, in a model of sepsis-induced intestinal barrier disruption (Wu et al., 2017), the P2X7 receptor antagonist showed a significant increase in the expressions of the tight junction proteins, occludin, claudine-1, and ZO-1 (Wu et al., 2017). The same study also showed that Bz-ATP (a purinergic agonist with potency for the P2X7 receptor) significantly decreased the expression of the tight junction proteins (Wu et al., 2017).

Our *ex-vivo* model has demonstrated a significant reduction of α-SMA in the myofibroblasts in the suburothelium following acrolein treatment. The myofibroblasts are spindle-shaped cells in the suburothelium, located just beneath the urothelium (Gevaert et al., 2012), and they can be stained for α-SMA as well as vimentin, desmin, and myosin (Powell et al., 1999). They usually are in close contact with the sub-urothelial afferent nerve terminals, suggesting their role in the activation of the sensory afferent nerves (Wiseman et al., 2003). The sub-urothelial myofibroblasts are contractile and have a regulatory role in the spontaneous or stimulated contractions of the mucosa layer (Sadananda et al., 2008; Cheng et al., 2011).

Our model has also shown that acrolein completely abolished the contractility of the mucosal strips to ACh and significantly diminished the contractility of the intact strips, with no significant change to the contractile response in the detrusor muscle. These results suggest that acrolein may severely damage the myofibroblasts and other contractile apparatus in the mucosa, but the perfusion time was not enough to damage the detrusor layer as the exposure to acrolein was from the luminal surface and the detrusor layer was protected by the mucosa. However, in the bladders pre-treated with A804598, both the contractile response to ACh and the sub-urothelial myofibroblasts were remarkably protected from the acrolein-induced damage.

Disrupted urothelium function, loss of the urothelial layer, and decreased urothelial tight junction expression are all characteristics of both IC/BPS patients and animal models of hemorrhagic cystitis (Slobodov et al., 2004; Graham and Chai, 2006; Bjorling et al., 2007; Hurst et al., 2015; Montalbetti et al., 2015). Similar to the findings from the current study, an *in-vivo* rat model of hemorrhagic cystitis induced by cyclophosphamide has shown that the urothelium was protected from damage and inflammation by the blockade of the P2X7 receptor activity (Martins et al., 2012). The mechanism behind this protection is not fully understood; however, ATP seems to be the connecting point. ATP acts as a neurotransmitter and signaling molecule, in that it binds to and activates purinergic receptors (Burnstock, 1972). A significant elevation in stretch-evoked ATP release has been demonstrated in the inflammatory conditions of the urinary bladder (Sun et al., 2001; Birder et al., 2003; Sun and Chai, 2006) as well as in the cyclophosphamide-induced hemorrhagic cystitis in rats (Smith et al., 2005). In the urinary bladder, all the purinergic receptors are expressed and, among them, the P2X7 receptor is mainly expressed on both the urothelium and the detrusor muscle (Lee et al., 2000; Vial and Evans, 2000; Menzies et al., 2003; Svennersten et al., 2015).

*Ex-vivo* models have been previously used to study the function of different organs such as the intestine (Jafari et al., 2016) and the eye (Chan et al., 2014). Compared to *in-vitro* models, such as cell culture, *ex-vivo* models allow for the examination of a more complex system and maintain the functional cell to the cell interactions that would usually occur within an organ (Pearce et al., 2018). Also, *ex-vivo* models provide an opportunity to study the pathophysiology and the function of an organ without pain and suffering of animals utilized in an *in-vivo* model. Our *ex-vivo* bladder model provides the opportunity to use the porcine bladder, which shows anatomical, physiological, and histological similarities to the human bladder (Kumar et al., 2004). The porcine bladders are also similar to the human bladders in terms of their urodynamic properties (Crowe and Burnstock, 1989; Moore and Brading, 2007). In contrast, the smaller size of the rodent bladders makes them different from their human counterparts regarding the baseline physiological properties (Parsons et al., 2012).

There are limitations in our study. It should be mentioned that a higher concentration of acrolein, compared to its presence in the bladder as the cyclophosphamide metabolite, was used in this study, enabling us to induce significant urothelial damage in a short exposure period and more importantly to investigate the protective effect upon the P2X7 receptor blockade. Another limitation of this model was the absence of communication between the urothelium and the nervous system, making it improbable to examine the interaction between the acrolein-induced urothelium damage and the inflammatory pain.

Mesna is an adjuvant used clinically with cyclophosphamide to reduce hemorrhagic cystitis. However, a recent paper reviewed 718 patients’ cases and found that the mesna group had a greater incidence of cyclophosphamide-induced hemorrhagic cystitis compared to the non-mesna group (Almalag et al., 2021). Our study demonstrates the protective effect of the P2X7 receptor inhibition on the urothelium in an *ex-vivo* acrolein model of bladder cystitis. The findings from this study provide evidence for the use of the P2X7 receptor antagonists for treatment of the bladder inflammation. These agents could potentially be co-administered with cyclophosphamide in the patients who undergo chemotherapy to reduce the use-limiting side effects associated with this treatment.

## Data Availability

The raw data supporting the conclusion of this article will be made available by the authors, without undue reservation.

## Abbreviations

α-SMA, alpha smooth muscle actin; DAB, 3,3′-diaminobenzidine; H&E, hematoxylin and eosin; IC/BPS, interstitial cystitis/bladder pain syndrome; ROS, reactive oxygen species; TBS-TX, Tris-Buffered Saline-Triton X; ZO-1, zona occludens-1; 8-oxo-dG, 8-hydroxy-2′-deoxyguanosine.
