# Supplementary material for: P2X7 Receptor Blockade Protects Against Acrolein-Induced Bladder Damage: A Potential New Therapeutic Approach for the Treatment of Bladder Inflammatory Diseases
Source: Front Pharmacol. 2021 Aug 12;12:682520. doi: 10.3389/fphar.2021.682520 (PMC8397461; doi:10.3389/fphar.2021.682520)
Supplement: Supplementary file 1 [file DataSheet1.PDF]

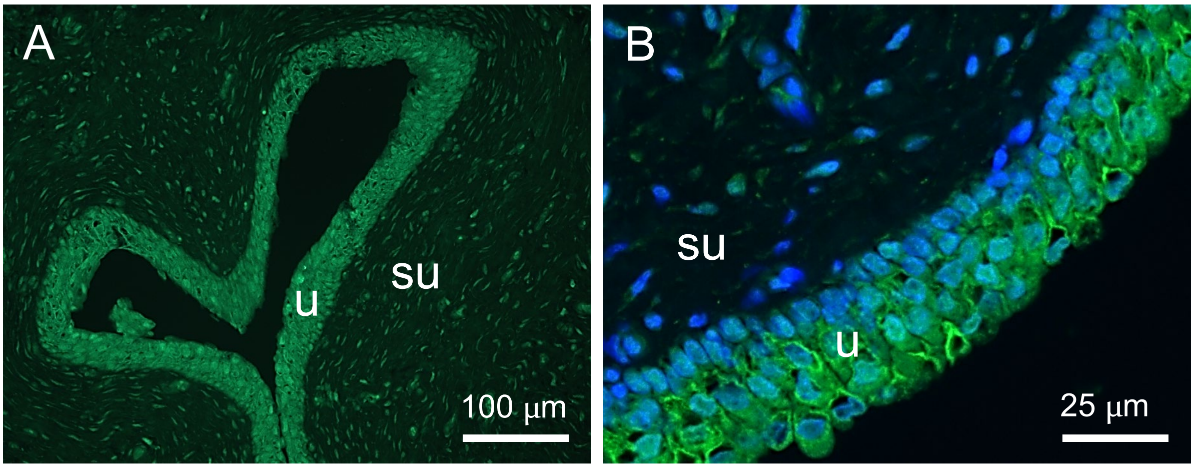

Supplementary Figure 1. Fluorescence immunohistochemistry of P2X7 receptor (green) in porcine bladder tissue with (**A**) 10 × and (**B**) 40 × magnification. Urothelium (U), suburothelium (SU) were labelled and DAPI (blue) is the nuclear marker. P2X7R antibody (1:100 dilution ab93354, Abcam).

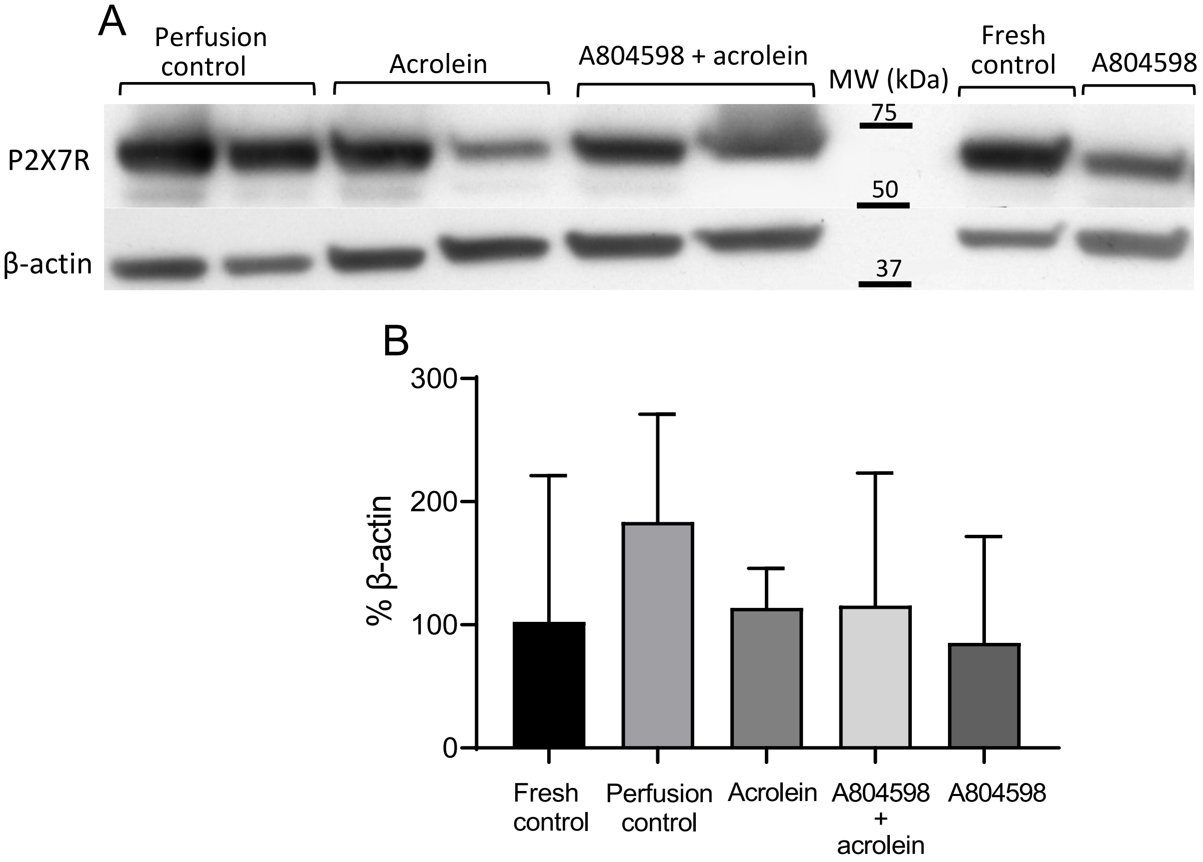

Supplementary Figure 2. Western blot and densitometry analysis of P2X7 receptor protein expression in the mucosal layer of porcine bladder collected from *ex-vivo* experiments. A). A single band for P2X7 receptor (~68 kDa) and β-actin (~42 kDa) corresponding to their expected molecular weight (MW) was observed. B). P2X7 receptor expression levels in different treatment groups are expressed as a percentage of β-actin, a loading control. Densitometry analysis was performed using ImageJ. One way ANOVA analysis between fresh control, perfusion control, acrolein (0.05%), acrolein (0.05%) + A804598 (10 μM) and A804598 (10 μM) was performed and there was no significant difference among groups. Data are expressed as mean ± SD (n = 6 - 9).

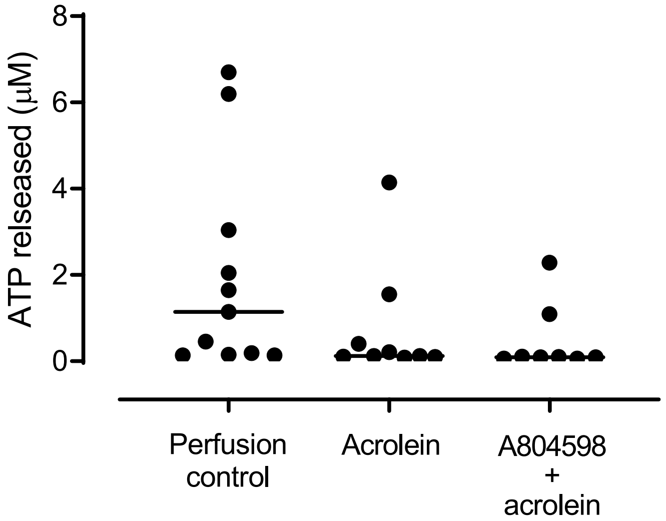

Supplementary Figure 3. ATP release in samples collected from the lumen of porcine bladders from *ex-vivo* experiments after 4 hours perfusion. ATP concentration in the perfusion media was measured using the ATP Bioluminescence Assay Kit (FLAA, Sigma-Aldrich) There was no significant difference between treatment groups. Data were expressed as scatter dot plot with the bar indicating the median (n = 8 -11, one-way ANOVA).
